# Contribution of students in the evaluation of master’s and doctoral programs in the field of nursing*

**DOI:** 10.17533/udea.iee.v40n1e01

**Published:** 2022-03-28

**Authors:** José Erivelton de Souza Maciel Ferreira, Lídia Rocha de Oliveira, Tahissa Frota Cavalcante

**Affiliations:** 1 Nurse, Master’s Student in Nursing. Email: eriveltonsmf@gmail.com Universidade da Integração Internacional da Lusofonia Afro-Brasileira Brazil eriveltonsmf@gmail.com; 2 Nurse, Master’s Student in Nursing. Email: lidiarocha2021@gmail.com Universidade da Integração Internacional da Lusofonia Afro-Brasileira Brazil lidiarocha2021@gmail.com; 3 Nurse, Ph.D. Adjunct Professor. Email: tahissa@unilab.edu.br Universidade da Integração Internacional da Lusofonia Afro-Brasileira Brazil tahissa@unilab.edu.br; 4 Universidade da Integração Internacional da Lusofonia Afro-Brasileira, Brazil. Universidade da Integração Internacional da Lusofonia Afro-Brasileira Universidade da Integração Internacional da Lusofonia Afro-Brasileira Brazil

**Keywords:** health postgraduate programs, education, nursing, graduate, teaching, higher education institutions, nursing.

Modern nursing has gained more space and scientific recognition. As a result, health postgraduate programs, including postgraduate education in nursing, are being increasingly expanded and implemented in numerous universities, higher education institutions, public and private colleges and institutes in several countries of the world. At the same time, more nurses have sought qualification in the field of teaching, research and professional practice, thereby resorting to master's and doctoral programs, specialization programs and MBA courses. Master’s and doctoral programs are the most sought after in the world nowadays. This demand has required from bodies responsible for education that these programs find new ways of qualifying the training of professionals who decide to ascend in the academic career. Therefore, having broken the banking model of education in higher education in most countries of the world, postgraduate students started to contribute directly to the qualification of these programs, even though their contribution still needs more recognition and seriousness in educational institutions.

The liberating perspective of education defended by Paulo Freire(1) was fundamental for the recognition of the student as an agent capable of improving the classroom itself, as it broke the hierarchical logic between those who transfer and those who receive knowledge, prioritizing horizontality in this relationship. As this perspective of education began to be mirrored and used as a basis for postgraduate teaching in many countries, there was a greater acceptance of the postgraduate student as an active participant in the evaluation of these programs - especially in the countries of America, Africa and Europe, continents where Freire’s pedagogical proposal is adopted in teaching.

In the last two years, the scenario imposed by the COVID-19 pandemic forced these higher education institutions to reorganize their ways of operating.(2) In addition, they also had to review their forms of evaluating the quality of their postgraduate programs. Such changes were not only intended to accompany the emergency health measures imposed, but also to continue the development of their teaching, research, extension, management and care activities with excellence.(3) This new pandemic scenario has placed expectations on these programs, especially regarding their students’ scientific and technical contributions concerning this calamitous public health problem.(4) Therefore, embracing and discussing the demands of this public is necessary and important, even to generate new reflections, for example, on the entry of postgraduate students in Latin American countries.

Note that a low percentage of people in the world manage to enter higher education, and of these, few choose or have access to an academic career through master’s or doctoral studies.(5) In a more global analysis, this results from several factors and conditions permeating inequalities of social class, gender and race, even in a context of educational expansion.(4) Allied to the aforementioned factors and conditions, is the fact of not publicizing the relevance of postgraduate programs for the professional and academic career of professionals since undergraduate studies. These aspects can negatively interfere in students’ critical and analytical capacity, which is a quality of a good researcher attending a program that needs his/her contribution to be improved.

In view of this, scientific production and thinking must be stimulated during undergraduate studies, so researchers can perform better in postgraduate studies, especially in academic master’s and doctoral programs. This incentive is justified because postgraduate students produce science and technical materials, and the survey of their production throughout the program is an important quality assessment criterion to be considered. All these aspects are essential to consolidate these subjects’ training and maintain the quality of programs.(6)

However, professionals who choose to enter a postgraduate program should not only emphasize their academic-professional profile based on ethical-aesthetic-political attitudes, or by their willingness to fulfill the mission and philosophy of the program and produce science within his area. Their role as students of the program is also to seek solutions and answers to the various critical nodes surrounding educational institutions themselves from a political, social and economic perspective, as well as their influence on the development of master’s and doctoral students. These subjects’ involvement in technical production and their contribution to social responsibility and innovation are necessary.

Over the years, postgraduate programs had to seek means of self-evaluation to correct existing flaws in their operationalization, and the instances above them had to guide strategies and methods to properly evaluate them.(7) Since students are part of the program, they are relevant in this process. Their contributions can innovate and expand curricula and the formation of interests that favor the program and the interests of student themselves and their advisors, which has been strongly perceived in the current pandemic scenario. Thus, taking the participation of students seriously in the evaluation of these programs will contribute to a more realistic evaluation of their processes, procedures, instruments and results. The dedication of students to the program reflects not only in their professional prominence, but also in the growth of the program and the institution to the point of becoming a reference educational establishment.

Students have the know-how to evaluate their postgraduate programs and the right to seriousness and respectful listening especially regarding the following demands: the faculty, management and the dynamics of operationalization of the program. Undoubtedly, this type of evaluation forms and consolidates a highly qualified program. Positive rates related to the quality of the program contribute substantially to achieve greater federal, state and municipal financial incentives for financing relevant, transformative and high-impact research of students.

Since the last century, master’s and doctoral programs in nursing have sought to overcome the Cartesian paradigm still present in the academic environment, which is based on the assumption that in order to know the whole, it is necessary to fragment it.(8) With this in mind, students need their guaranteed inclusion in committees dedicated to managing the evaluation processes of these programs. This is necessary to continue breaking this paradigm that is still present in so many internationally renowned institutions.

The training of master’s and doctoral students in nursing contributes to produce new forms of knowledge in the field of nursing and health sciences in general.(9) Therefore, the educational institution must recognize that these subjects are continuously immersed in a process of maturation of their social role as masters and/or doctors in the academic, scientific and sociopolitical environment hence, they can directly contribute to the evaluation of these programs. Given all considerations presented, listening to their demands is important, urgent, to improve the quality of these programs in terms of strategic planning for teaching and management.
